# Lessons from the removal of lead from gasoline for controlling other environmental pollutants: A case study from New Zealand

**DOI:** 10.1186/1476-069X-7-1

**Published:** 2008-01-07

**Authors:** Nick Wilson, John Horrocks

**Affiliations:** 1 Department of Public Health, University of Otago, Wellington, Box 7343 Wellington South, New Zealand; 2 School of Health and Wellbeing, Wellington Institute of Technology, Private Bag 39803, Te Puni Mail Centre, Buick Street, Petone, New Zealand

## Abstract

**Background:**

It took over two decades to achieve the removal of leaded gasoline in this country. This was despite international evidence and original research conducted in New Zealand on the harm to child cognitive function and behaviour from lead exposure.

**Objective:**

To identify lessons from the New Zealand experience of removing leaded gasoline that are potentially relevant to the control of other environmental pollutants.

**Discussion:**

From the available documentation, we suggest a number of reasons for the slow policy response to the leaded gasoline hazard. These include: (1) industry power in the form of successful lobbying by the lead additive supplier, Associated Octel; (2) the absence of the precautionary principle as part of risk management policy; and (3) weak policymaking machinery that included: (a) the poor use of health research evidence (from both NZ and internationally), as well as limited use of expertise in academic and non-governmental organisations; (b) lack of personnel competent in addressing technically complex issues; and (c) diffusion of responsibility among government agencies.

**Conclusion:**

There is a need for a stronger precautionary approach by policymakers when considering environmental pollutants. Politicians, officials and health workers need to strengthen policymaking processes and effectively counter the industry tactics used to delay regulatory responses.

## Introduction

Lead has been described as "a public health problem of global dimensions" [1]. Children are much more at risk from lead exposure than adults because of higher rates of absorption of ingested lead and the harm in terms of impaired cognitive development and antisocial behaviour (reviewed by Tong et al and Fischbein [1,2]). Recent evidence suggests these effects can occur at low levels of exposure [3,4]. There is also a growing body of evidence that relatively low levels of lead exposure cause adverse effects on cardiovascular and renal function in adults [5].

The phasing out of lead from gasoline is considered to be a critical step in reducing population blood lead concentrations [6]. Public health initiatives to limit lead exposure have a long history [7] but the experience from the United States (US) and other countries indicates that the reduction and elimination of leaded gasoline has probably been the major factor in recent times in reducing population blood lead levels [8-10]. Given this concern about lead in gasoline, agencies such as the World Health Organization (WHO) and the World Bank have focused their efforts on reducing this particular source of lead [6]. The final stages of the elimination of lead additives in gasoline is now coming from a United Nation's "Partnership for Clean Fuels and Vehicles", launched in 2002. It has the goal of getting rid of leaded gasoline worldwide by the end of 2008 [11].

Lead was eventually removed from gasoline in New Zealand in 1996. However, the process of this particular policy development has never been studied. Our goal in this commentary is to identify lessons from a case study of this country that are potentially relevant to the control of environmental pollutants in other countries. To do this we undertook literature searches and examined archived official documents for the New Zealand setting. These covered the period 1974 (the year of the Clean Air Council's report [12]) to 1996, the year that unleaded gasoline became mandatory. Particular attention is given to the period before 1984, when the New Zealand Government made the initial decision to introduce unleaded gasoline.

## Discussion

It took over two decades to achieve the elimination of leaded gasoline in New Zealand. Table 1 highlights some of the key events and indicates that New Zealand, in particular, was fortunate to have relatively high quality scientific evidence on the hazard posed by lead to child health. Nevertheless, it took eight years from the time that key local child health research was first published (in 1988) to the final phase-out date for leaded gasoline in 1996. The lag between the US Environmental Protection Agency's (EPA) first regulatory moves against leaded gasoline in 1972, to the start of New Zealand's phase-down in 1986, was 14 years (Table 1). In the following sections we detail some of the major elements which contributed to this drawn-out process.

**Table 1:** Chronology of the key scientific research and policy developments*

**Year/s**	**Developments**	**Reference**
Late 1800s	***Policy***: There is enough medical concern about the health effects of lead contaminated water for some US authorities to regulate the lead water piping hazard.	7
1909	***Policy***: Countries begin to enact bans or restrictions on the use of white lead for interior paint (eg, France, Belgium, and Austria in 1909). In 1922, the Third International Labour Conference of the League of Nations recommended the banning of white lead for interior use.	[72]
1923	***Leaded gasoline***: Tetraethyl lead begins to be sold for use in gasoline. Early production was associated with severe occupational health consequences and deaths among workers [73].	65
1970	***Industry policy***: The vehicle manufacturer General Motors announces that it would begin installing catalytic converters in its new models and that as a result it would be necessary to phase out lead in gasoline.	65
1972	***Japanese policy***: Japan becomes the first country to market unleaded gasoline and by 1981 less than 3% of gasoline sold there is leaded.	[74]
1972	***US policy***: The US EPA issues rules that each gasoline station has to have at least one lead-free pump. This is to protect the platinum catalytic converter on new model vehicles to facilitate air pollution control in general (and not primarily to protect humans from lead exposure).	[65]
1973	***US & Canadian policy***: After a growing body of evidence for adverse health effects from lead toxicity [65], the US EPA announces new regulations to restrict the content of lead in gasoline. Unleaded gasoline was also introduced in Canada in the same year (1975) [74].	[75]
1973	***NZ advocacy***: The Environmental Defence Society petitions the Clean Air Council on the topic of leaded gasoline. It is the first non-governmental organisation to campaign against lead additives [Dr Robert Mann, Personal communication, 27 September, 2007].	
1974	***NZ policy advocacy***: The Clean Air Council (a government advisory body) calls for a phase-down of lead in gasoline. At this time the lead level was the highest in the world at 0.84 g/L [76]. Also in this year regulations were introduced in NZ that aimed to eliminate lead solder from canned foods and beverages [77].	[12]
1975	***NZ research***: The first systematic study into lead exposure among children reports relatively high soil lead levels from the roadsides of busy urban streets.	78
1978	***NZ research***: Elevated levels of lead are found in the blood of sheep grazing beside a rural highway.	79
1981	***NZ policy advocacy***: The Toxic Substances Board resolves on 7 August that "...on the grounds of health hazard to some significant proportion of the population..." lead additives be deleted from gasoline as soon as practicable.	31
1983	***NZ policy advocacy***: The Ministry of Energy issues a white paper on lead in gasoline, providing costs for different lead-reduction scenarios and suggesting no policy steps should be taken until the need was demonstrated by a population blood-lead survey. This year also saw the Toxic Substances Regulations that restricted the content of lead in paints used on furniture, toys, and household items [77].	13
1984	***International/NZ research***: A multi-country study that includes NZ data indicates higher levels of lead in street dust from a busy road relative to a road in a "quiet suburban area".	80
1984	***NZ research review***: A comprehensive workshop at Auckland University brings together most NZ researchers on lead and other interested parties in order to plan future research programmes.	81
1984	***NZ policy***: The incoming Labour Government announces on 3 August that unleaded gasoline will be available from 1986 when the extensions to the country's sole refinery are completed and a synthetic gasoline plant is operating.	82
1985	***NZ research***: A study which examined house dust in a city reports "for the newer areas of the city, it was estimated that ~90% of the lead was derived from gasoline additives (via street dust and aerosol)...".	83
1986	***NZ advocacy and policy***: An analysis jointly sponsored by the Government and the Royal Society of NZ strongly urges the phase-down and eventual elimination of gasoline lead. The international evidence on costs to health is acknowledged by the Ministry for the Environment in an options paper. The start of the lead phase-out begins in this year.	[84,85]
1986	***International/NZ research***: A NZ scientist publishes a detailed review article on the contribution of leaded gasoline to blood lead levels. It stated that: "the weight of evidence suggests that at least one-third of blood lead comes from petrol lead".	39
1986	***NZ research***: Further longitudinal study data identifies blood lead levels in children and describes some of the concerns in the international literature about possible associated cognitive deficits and behaviour problems.	86
1987	***NZ action***: Leaded 91 (RON) octane gasoline is replaced by unleaded 91-octane gasoline. Higher-octane (96RON) gasoline remains leaded.	
1988	***NZ research***: Results from a longitudinal study show that blood lead levels were associated with a statistically significant increase in children's general behaviour problems.	87
1988	***NZ research***: A study shows that the potential benefits of reducing leaded gasoline (eg, reduced engine wear and opportunity to use the latest engine technology) are likely to exceed any cost involved in conversion to a wholly unleaded fuel supply by 1996.	46
1988	***NZ research review***. A workshop on the impact of lead in the NZ environment is held.	88
1988	***NZ research***: Publications from another longitudinal study implicate various risk factors for raised lead levels, including residence near busy roads. This work reports statistically significant negative correlations between dentine lead values and all measures of school performance and ratings of inattention/restless in children.	[89-91]
1989	***International research***: A summary of a report to the US Congress on childhood lead poisoning documents the relative persistence of adverse effects from neurotoxicity and on growth.	92
1990	***International research***: A meta-analysis concludes that "the hypothesis that lead impairs children's IQ at low dose is strongly supported".	93
1991	***International/NZ research***: A review of NZ and international data reports how lead in house dust is associated with distance from roads and also the road type (ie, traffic density).	94
1992	***NZ policy***: Ministry for the Environment releases air quality guidelines.	95
1993	***NZ research***: This research reports that early exposure to lead resulted in statistically detectable and apparently enduring deficits in cognitive abilities, poorer school performance and behavioural problems.	[96,97]
1993	***NZ policy review***: The Ministry of Commerce produces a paper on required changes to regulations to facilitate the introduction of unleaded gasoline.	98
1994	***NZ research***: The sources of lead in the atmosphere of the urban environment are estimated to be mainly automobile emissions.	99
1995 – 1996	***NZ policy***: The Minister of Energy announces that no leaded gasoline will be able to be sold after 30 September, 1996.	100
Post 1996	***NZ & international research***: Reports detail the reduction of lead in gasoline over the period in the decade after 1986 in NZ and associate it with declines in atmospheric lead levels and declines in population blood lead levels (ie, data for between 1984/5 and 1994 for declining blood lead levels in Christchurch residents [10]). There is also evidence for continuing reductions of blood lead levels of occupational groups involved in vehicle repairs since the lead phase-out began in 1987 [77].	[10,101]

* For developments concerning the hazard of lead exposure with particular reference to leaded gasoline and to New Zealand.

### 1) The lack of a precautionary approach

The overriding approach to the issue of leaded gasoline in New Zealand was one of delay and evasion by government agencies. This was frequently stated as being on the grounds that it was first necessary to establish that there was really a hazard. We found little evidence that officials were sympathetic to a precautionary approach. That is that the evidence of risk suggested child health should be protected even if the precise dimensions of the exposure levels and harm to cognitive and behavioural functioning had still to be established.

An illustration of these attitudes was the argument by the country's Chief Air Pollution Control Officer and the Ministry of Energy, supported by the suppliers of lead additives (Associated Octel), that a survey of blood lead levels in the New Zealand population was needed before decisions could be made on lead reduction. This was despite existing New Zealand evidence suggesting a hazard and the evidence that agencies in other countries had already decided lead additives were harmful and had acted to reduce their impact (eg, the US in the 1970s – see Table 1). Particularly unusual was the fact that the protocols for this proposed national study, with its implications for public health, were initiated by the Ministry of Energy, which even suggested what a desirable mean blood lead level in the population might be [13]. Even when there was subsequently evidence for adverse impacts on children from a longitudinal study in New Zealand (see Table 1), this appeared to have little or no impact on the policy process.

An early review of the New Zealand situation noted the wealth of international evidence upon which policymakers could already draw [14]. This was enough to give the benefit of the doubt to children rather than the lead additive industry. Others at this time were also advocating the abolition of leaded gasoline even though the evidence for harm still involved uncertainties (eg, the *British Medical Journal *[15] and researchers in neighbouring Australia [16]).

The implementation of a precautionary principle in integrated risk management in New Zealand is now under consideration by Treasury [17]. If such a policy had been in place in the 1980s, different decisions might have been taken about a number of measures that would have reduced lead emissions. These could have included: (i) requirements for all new cars to run on unleaded fuel after unleaded gasoline became available in 1986 [18]; (ii) stronger encouragement of unleaded transport fuels such as compressed natural gas and liquefied petroleum gas; (iii) and support for alcohol blends in gasoline as an alternative route to high-octane fuel without lead additives. The latter option was researched in several studies by the Liquid Fuels Trust Board in the 1980s and there were even trial pumps installed at filling stations [19].

New Zealand has only one oil refinery, operated by the New Zealand Refining Co Ltd. The absence of a precautionary principle was repeatedly demonstrated in its decisions (in consultation with government), regarding the form of successive refinery expansions. The capacity to produce enough unleaded gasoline to supply the bulk of the local market was reduced by the decision to base the refinery modifications in the early 1980s around a hydrocracker, rather than a catalytic cracker [20]. Later decisions also limited the refinery's capacity to produce unleaded gasoline [18]. However, there was also limited flexibility as the result of the key role the refinery played in the supply of other products for the New Zealand market, such as diesel. When subsequent modifications were required in the 1990s to produce high-octane (96RON) unleaded gasoline, these were held up, in turn, by a lack of clear direction from government. This frustrated refinery management, who by this time just wished to know what the politicians wanted and described the government as "floundering" [21].

### 2) Weak policymaking machinery

In addition to the lack of a precautionary approach, there is evidence that other deficiencies in the policymaking machinery of government contributed to the delay in adopting measures to reduce lead exposure. This was in part because the officials and politicians were not effective in countering industry arguments (as detailed subsequently) but other factors included: (i) poor use of the health research evidence (from both NZ and internationally), as well as use of the expertise in the New Zealand research community; (ii) lack of personnel competent to address technically complex issues; and (iii) the absence of a single government agency which had overall responsibility for the issue.

#### (i) Poor official use of research evidence and the research community

The evidence for the widespread presence of lead in the New Zealand environment first appeared in the 1970s [22]. Levels of lead in air in central locations in Christchurch and Auckland were consistently found to exceed US EPA standards of 1.5 μg/m^3 ^over a three-month period [23,24]. Then in the 1980s there was evidence from two different longitudinal studies for an adverse impact of lead on child health (Table 1). Yet even with such evidence there was little use of this information by officials to highlight the threat to child health. Furthermore, when New Zealand research did receive official comment, this comment could be not only misleading but also imply a lack of understanding of the data. An example came in 1980, in a reply to a written question in Parliament. The Minister of Health, George Gair, said that the blood lead levels of children living near the Southern Motorway in Auckland were no different from those of children living in three other locations [25]. His advisers had failed to note that the researchers, from the Auckland Medical School, had found a significantly lower mean blood lead level in a reference group of children living in a new estate on land that had previously been rural. Besides the group living alongside the motorway, the other groups in the study included one of children from an inner-city suburb of old, lead-painted houses and another who lived near a lead battery factory [26]. This study suggested, at the very least, that environmental variations might account for the differing levels of lead found in the blood of these children. It hardly justified the Minister's assertion that: "What is clear is that lead in petrol is not likely to be responsible for high blood lead levels in children."

New Zealand authorities discounted the relevance of international research by their continuing insistence that New Zealand was relatively free of air pollution, or well "ventilated", as one put it [27]. In 1987, the Chief Air Pollution Control Officer for the Health Department asserted that the density of motor cars per square kilometre was low in New Zealand, thereby implying that motor vehicle pollution was of limited significance [28]. This view completely ignored the high *urban *density of vehicles. There was a similar response to Labour Party Member of Parliament (MP), Philip Woollaston, when he attempted to legislate for unleaded gasoline in 1982. His private member's bill, the Lead Pollution Control Bill, was received with scorn by members of the governing National Party. He was told that the bill was an example of "emotional bandwaggoning" and that "New Zealand has a remarkably good atmosphere, noted for the purity of its air and the clarity of its light" [29]. This attitude is still commonly encountered, despite evidence that far more people die each year in New Zealand as the result of air pollution from vehicles than from motor vehicle crashes [30]. These rosy evaluations also overlooked the 1981 recommendation by the Toxic Substances Board, a Department of Health advisory body, that lead be removed from gasoline as soon as was practicable on the grounds of a risk to health [31].

This history of neglect meant the decision to introduce unleaded gasoline, announced by the newly-elected Labour Government in August 1984, was unusual by international standards in that it did not follow the type of considered policy evaluations that preceded decisions on this issue in Australia and the United Kingdom. It was very much a political decision. This was reflected in continuing divisions of opinion between government ministers themselves, and the persistence of scepticism by Minister of Energy Bob Tizard, in particular, about the need for lead reduction [32].

In 1986 the Royal Society of New Zealand (RSNZ) recommended that the Government should explore means to eventually remove all lead from gasoline, while ensuring that by 1990 premium grade gasoline contained no more than 0.15 g/l of lead [33]. It also observed that it would be fraudulent to advertise New Zealand as having clean air. Despite this advice, the Minister still cast doubt in the following month (July 1986) over whether it would be necessary to reduce the lead content further in the higher 96-octane grade. This, he said, would depend on whether research showed such a move was necessary [34]. Subsequent similar comments and the observation that this was a "very emotional issue" [32], drew a sharp response from the scientific community. Dr Gail Irwin, speaking for the New Zealand Association of Scientists, observed that the Minister's remarks were inconsistent with the findings of the report by the Royal Society that he himself had commissioned [35].

#### (ii) Weak technical capacity

The capacity within the Department of Health for independent analysis of the issues about leaded gasoline and vehicle fuel alternatives issues appears to have been very limited, especially in the period up to 1984. There were no staff with specific training in environmental health epidemiology, though some staff had training in toxicology (eg, the Senior Toxicologist which was a position that provided technical advice to senior public health officials). There were also no government agencies with environmental epidemiologists upon which the Department could call upon to obtain advice. Yet despite such lack of expert advice, the Department's position was definite and straightforward: there was no need for a rapid move towards unleaded gasoline. This view was publicly expressed by the Director of Public Health [36], the Deputy Director [27], and the Department's Chief Air Pollution Control Officer [28].

A key technical issue during the debate was the respective contributions of different sources of lead exposure. Some researchers [37] focused on non-gasoline sources of lead and amongst officials and politicians there was a persisting view that lead additives were of minor importance. The Chair of the 1986 RSNZ Report on lead noted the differing views held between the Royal Society scientists and officials in the Department of Health and the Ministry of Energy (describing the latter as "doctrinaire") [38]. In 1983, for example, the Ministry of Energy's white paper, *Lead in Gasoline *had stated that, "There is no strong relationship overseas between lead levels in gasoline and lead levels in blood" [13]. In 1986 the Minister of Energy went even further in claiming that there was no proven link between lead in gasoline and lead in people in New Zealand [32]. In stark contrast to this position, a review in the same year (by a New Zealand scientist) concluded that a third of blood lead came from lead additives [39].

The Ministry of Energy's viewpoint was endorsed by the Australasian Manager of Associated Octel, who described *Lead in Gasoline *as "a most commendable document" [40]. An apparent lack of capacity for analysis independent from the information provided by the oil and lead additives industries was perhaps most clearly illustrated in the early 1980s by the fact that government officials in the Oil Marketing Directorate depended upon the New Zealand Refining Company's computer for modelling options for lead reduction [41]. A lack of technical capacity or purpose amongst officials may also explain why there was so little urgency to establish comprehensive regulatory controls relating to vehicle emissions other than lead. Indeed, it was not until the period 2003–2006 that New Zealand moved decisively in this direction, principally through requirements that imported vehicles should meet country of origin emission standards [42].

#### (iii) Divided responsibility for decisions

By the time that all leaded gasoline was finally withdrawn from the New Zealand market, numerous government departments or advisory bodies had had some involvement in the process. These included the Ministry of Health (formerly the Department of Health), the Commission for the Environment, the Ministry for the Environment, the Ministry of Commerce, the Ministry of Energy, the Ministry of Science and Research, the Clean Air Council, and the Toxic Substances Board. In 1984 the Government also commissioned the Royal Society of New Zealand to report on lead in the environment. Unfortunately, however, there was no single agency (such as the Ministry of Health or the Ministry for the Environment) that took a strong precautionary and public health approach to the issue, despite detailed proposals about how an integrated pollution control strategy of this kind could have been developed [43]. This diffusion of responsibility took on comic-opera dimensions at times. By 1990 it was hard for reporters to find out which agency was actually responsible for the lead phase-down: the Ministry of Energy, the Ministry for the Environment, or the Ministry of Commerce [44].

Government ministers were often reluctant to take decisive action themselves, expressing the hope that the market participants (ie, industry and consumers, in their view) would work towards lead elimination without the need for government regulation [45]. This wish was to prove naïve, with regulation being eventually required. One consequence of such attitudes was a loss of expertise when the Ministry of Energy itself was downsized in the late 1980s, which made it more difficult for officials in government agencies to evaluate decisions such as how best to expand New Zealand's oil refinery [44].

In the final stages of the debate it became apparent that developments in engine design meant that New Zealand, by sticking with leaded gasoline, would be unable to take advantage of new engine technologies and would pay a fuel penalty as the result. This was recognised in a cost-benefit analysis prepared for the Ministry for the Environment in 1988 [46]. Engineering and cost considerations were thus to prove critical in driving the 1996 final phase-out at a time when the health issues were still being contested. There was never an attempt to quantify the health costs of using leaded gasoline in New Zealand.

### 3) Industry power

The industry players in the New Zealand setting in the 1980s and 1990s were the manufacturers of the lead additives themselves, Associated Octel (a British company), the operators of New Zealand's only refinery, the New Zealand Refining Company (NZRC), the oil companies, the Motor Vehicle Manufacturers Association, and the Motor Trade Association. The lead additives supplied to NZRC came from Associated Octel. The principal shareholders in NZRC were the oil companies that operated in New Zealand and these companies were, in their turn, major shareholders in Associated Octel. Though this commercial relationship was close, the oil companies themselves kept at arms length from the controversy over lead additives and it was left to Octel itself to fight to retain its market.

#### Relationships with officials

Octel worked hard to establish a good relationship with officials in the Ministry of Energy. For example, the company expressed its gratitude to the head of the Ministry's Oil Market Directorate, Andre Milkop, for facilitating a series of discussions in July 1983 between Octel staff and representatives of various government ministries, as well as the Minister of Energy (Bill Birch) and the Minister for the Environment (Dr Ian Shearer). The closeness of the association between Milkop and Dr David Gething, Octel's Chief Medical Officer at the time, was suggested by Gething's letter to him in which he asked for Milkop's advice about the mood in the Ministry for the Environment: "I hope by now members of Dr Shearer's Department have had the opportunity to read all the information I left with them.....but if you could give me a steer on the feelings of the Environmental Department I should be most grateful" [47].

The material provided by Octel was comprehensive: reprints of articles from scientific journals, technical reports on emission technology, including Octel's favourite technical fix, a lead trap on the exhaust, rebuttals of reports such as that of the United Kingdom's 1983 Royal Commission on Environmental Pollution [48], and news clippings. There were also Octel's analyses of the health impact of lead in gasoline, such as Gething's glossy booklet on the issue [49]. More significant, however, was the thrust at this time by Octel to persuade the New Zealand authorities to change their air monitoring procedures in order to produce less alarming data. For example, Gething suggested in a meeting with Roger Holden, the Department of Health's Chief Air Pollution Control Officer, that air monitoring stations should not be sited by busy road junctions. This advice was followed up in a letter to Holden, in which Gething noted that these monitors "... give high lead-in-air figures and these are the ones that receive the most publicity and cause unnecessary alarm" [50]. The fact that some people actually live or work at such locations, was not mentioned.

The closeness of the relationship between the lead industry and New Zealand officialdom was noted by Dr Clair Patterson, from CALTECH, who was an authority on the historical exposure of humans to lead in the environment [51]. After a visit to New Zealand in 1983 (in which his views on lead in the environment were derided by the Christchurch Medical Officer of Health), Patterson subsequently wrote that there appeared to be "... an unhealthy liaison between the lead alkyl industries and public health officials in New Zealand... I was struck by the remarkable similarity of the New Zealand health official's attitude and arguments with those of officials of the Ethyl Corporation and Associated Octel" [52].

#### Countering expert advice

Octel was systematic in its attempts to nullify unfavourable impressions of its products. In 1981, for instance, the environmental group Friends of the Earth organised a tour by Australian chemistry professor, Dr Lloyd Smythe, who had been one of the authors of an extensive study of lead and its effects on Australian children [53]. Smythe was surprised to find that, when he came to meet with Members of Parliament and Department of Health officials in late August, they had already been supplied with a detailed critique of his research.

#### Building alliances

As the introduction of high-octane unleaded gasoline drew closer, (which would mean all gasoline would be unleaded), Octel sought out new allies. It predicted, for instance, that older cars would be consigned to the scrapheap, which would suit sellers of new cars, who were frustrated by the pride some owners had in their classic vehicles. This went down well with a writer for the magazine, *New Zealand Classic Car*, who challenged the "doomsayers", arguing that only 10% of lead came from the air [54]. In another car magazine, "DBO" mused that the information supplied to him by Octel suggested there might be "... a hidden agenda of some sort behind the push for the introduction of high-octane unleaded fuel in this country" [55]. He accordingly suggested a readers' FAX ATTACK to the Minister of Energy.

#### Scare tactics

Even in the final stages of the lead phase-down, Octel initiated a strong campaign to stress the health risks of high-octane (96 RON) unleaded gasoline, which was expected to produce a higher level of benzene in exhaust gases (as the result of chemical changes to the aromatics in the higher-octane fuel). This included a series of scare-mongering newspaper advertisements in New Zealand and other countries which stressed the carcinogenic effects of this increased benzene production [56]. Headed 'Unleaded petrol and cancer,' the advertisements equated high-octane unleaded gasoline with asbestos and cigarettes [57]. Lead was described as "a naturally occurring toxin, as are alcohol, sugar, and salt."

Commentary from consultants to Octel, and their visits to New Zealand (eg, [58,59]) appears to have contributed to concerns about benzene with several media reports around this time suggested that the move to unleaded gasoline was a policy blunder [60]. Not surprisingly, the industry ignored the existing source of likely carcinogens in leaded gasoline (according to the knowledge at this time). These were the "scavengers" ethylene dibromide and ethylene dichloride [61,62], added to leaded gasoline in order to help prevent a build-up of lead deposits in the cylinders. Such tactics may have had some political impact, in that in late 1994 the Minister of Energy started to vacillate on the leaded gasoline phase-out, citing concerns about benzene and saying that "...claims by interest groups that lead is less harmful than estimated need further examination" [63]. The interest groups were unspecified, but there seems only one clear candidate, Octel itself.

## Conclusion

From the available documentation, we identified three likely major reasons for the slow progress to phase out leaded gasoline in New Zealand. These included: (1) industry power in the form of successful lobbying by the lead additive supplier, Associated Octel; (2) the absence of a precautionary principle as part of risk management policy and (3) weak policymaking machinery. Given the complexity and importance of the issues, further analysis of the historical developments is clearly warranted, together with comparative analyses of the lead additives endgame in other countries. Further research to clarify the importance of these different reasons could be based on multiple key informant interviews and a more comprehensive examination of official documentation.

Nevertheless, some of the findings in this New Zealand case study are consistent with the delays observed in other countries that were acting to phase out lead additives [64,65]. Similar findings apply to responses to the hazards of lead piping [7] and leaded paint [66,67]. An extreme example of this was in France, where the dangers of lead in paint had to be more or less rediscovered in the mid-1980s [68]). These laggardly responses are familiar in instances of other environmental health hazards such as asbestos, second-hand smoke and greenhouse gases. In the case of second-hand smoke, for example, there have been lags of several decades from the time a hazard to health was identified and the enactment of regulations that adequately restrict smoking in public places (with the historical scientific evidence detailed in a Surgeon General's Report [69], and current laws detailed by Novak [70]).

The New Zealand experience illustrates the weakness in decision-making that comes when no single government agency akin to the US EPA has overall responsibility for an environmental health issue. This weakness should be addressed directly in the course of any initiatives to place a precautionary principle at the heart of policy formation. Strengthening policymaking processes for environmental health issues also requires appropriate in-house technical expertise in central government, adequate resourcing to resist industry pressure, and openness to input from the expertise in civil society provided by non-governmental organisations and universities.

Yet even with more appropriate structural arrangements and processes it cannot be assumed that EPA-like bodies will always work optimally to protect public health. The US EPA, for example, refused to consider lead a criterion pollutant until it was sued by National Resources Defense Council [65]. Even now the current US air lead standard is 1.5 μg/m^3^, three times that recommended by WHO.

For small countries such as New Zealand, the lack of appropriate government agencies to address such environmental health issues might be ameliorated in the future by drawing on technical support from international bodies such as the WHO. These bodies can also have the capacity to develop relevant international treaties such as the Framework Convention for Tobacco Control [71].

## Abbreviations

EPA: Environmental Protection Agency;

MP: Member of Parliament;

NZ: New Zealand;

NZRC: New Zealand Refining Company;

RSNZ: Royal Society of New Zealand;

US: United States;

WHO: World Health Organization.

## Competing interests

The first author (NW) declares no competing interests. The second author (JH) undertook paid work on the leaded gasoline issue for the non-profit non-governmental group Friends of the Earth (NZ) between 1979 and 1988.

## Authors' contributions

Both authors made substantial contributions to preparing this paper, including the design level, examining literature and in the writing of drafts. One of us (JH) did most the examination of the archival material. We give final approval of the version to be published. Both authors have participated sufficiently in the work to take public responsibility for appropriate portions of the content. Both authors have read and approved the final manuscript.
